# Carbapenem Resistance in Clonally Distinct Clinical Strains of *Vibrio fluvialis* Isolated from Diarrheal Samples

**DOI:** 10.3201/eid2210.151612

**Published:** 2016-10

**Authors:** Goutam Chowdhury, Gururaja Perumal Pazhani, Anirban Sarkar, Krishnan Rajendran, Asish K. Mukhopadhyay, Mihir K. Bhattacharya, Amit Ghosh, Thandavarayan Ramamurthy

**Affiliations:** National Institute of Cholera and Enteric Diseases, Kolkata, India (G. Chowdhury, G.P. Pazhani, A. Sarkar, K. Rajendran, A.K. Mukhopadhyay, M.K. Bhattacharya, A. Ghosh, T. Ramamurthy);; National Institute of Pharmaceutical Education and Research, Kolkata (G.P. Pazhani);; Translational Health Science and Technology Institute, Faridabad, India (T. Ramamurthy)

**Keywords:** V. fluvialis, Enterobacteriaceae, diarrhea, NDM-1, NDM-VF, PFGE, antibiotic, antimicrobial resistance, carbapenem, New Delhi metallo-β-lactamase, gram-negative bacterium, bacteria, Vibrio fluvialis

## Abstract

Carbapenems have been used for many years to treat severe nosocomial *Enterobacteriaceae* infections. The spread of resistance to these drugs among other bacterial families is an emerging problem worldwide, mostly caused by New Delhi metallo-β-lactamase (NDM-1). We screened for the prevalence of NDM-1–expressing enteric pathogens from hospitalized patients with acute diarrhea in Kolkata, India, and identified 27 *Vibrio fluvialis*–harboring *bla*_NDM-1_ (NDM-VF) strains. These isolates were also resistant to all the tested antimicrobial drugs except doxycycline. The large plasmid of *V. fluvialis* harboring *bla*_NDM-1_ could be easily transferred to other enteric pathogens. Genes flanking the *bla*_NDM-1_ were found to be identical to the reported sequence from an *Escherichia coli* isolate. Analyses showed that the *V. fluvialis* possessing the NDM-VF region belonged to different clones. The pathogenicity of *V. fluvialis* to humans and its ubiquitous presence in the environment call for constant monitoring of this species for emerging antimicrobial drug resistance.

## Abstract 2

These strains might acquire the *bla*_NDM-1_ gene without exposure to antimicrobial drugs.

The increasing incidence of carbapenem-resistant bacterial infection is a major public health concern (*1*). Several species of carbapenemase-producing bacteria also display co-resistance to most, if not all, available antibiotic drugs used against different infections, thereby limiting the medication options (*1*). The novel carbapenemase New Delhi metallo-β-lactamase (NDM-1), encoded by the gene *bla*_NDM-1_, has been identified in many pathogenic members of the family *Enterobacteriaceae*, which are capable of colonizing hosts and also transfer the *bla*_NDM-1_ gene region to other bacteria. Several of these bacteria have been associated with contaminated hands, food, and water in hospitals, community settings, and in the environment (*1*). However, reports on the prevalence of *bla*_NDM-1_ among enteric pathogens are relatively fewer.

NDM-1–producing *Klebsiella pneumoniae* was first identified in 2008 in a urine sample from a traveler from Sweden who acquired a urinary tract infection in India (*2*). Investigations by Kumarasamy et al. (*3*) led to the initial report of widespread prevalence of NDM-1 in *Escherichia coli* and *K. pneumoniae* strains isolated from several clinical settings in India, Pakistan, and the United Kingdom. Numerous studies in subsequent years reported NDM-1–producing *Enterobacteriaceae* and other bacteria, including *Vibrio cholerae* in many countries (*4*–*7*). Recently, several reports on carbapenemase-producing *Enterobacteriaceae* in India have been published (*8*–*10*). In unrelated gram-negative bacteria, the presence of the *bla*_NDM-1_ gene has been reported to be associated with several plasmid incompatibility types (e.g., IncA/C, IncF, IncL/M, IncH, or untypeable) or was found integrated into the chromosomes (*11*). Because the gene *bla*_NDM-1_ located on plasmids is also carrying bacterial growth promoter regions, the possibility of gene transfer to other gram-negative bacteria is very high (*12*). 

*V. fluvialis* is known to be commonly present in many aquatic environments and seafood (*13*). This organism has been reported as an emerging pathogen associated with cholera-like diarrhea in India and China (*14*,*15*). We report the identification and characterization of NDM-1–producing *V. fluvialis* strains isolated from diarrheal fecal samples from patients admitted to the 2 hospitals in Kolkata, India. 

## Materials and Methods

Using systematic active surveillance, we enrolled every fifth hospitalized patient at the Infectious Diseases Hospital (IDH) and B.C. Roy Memorial Hospital for Children (BCH) in Kolkata who had diarrhea or dysentery on 2 randomly selected days of the week during May 2009–September 2013. Diarrhea was defined as >3 episodes of loose or liquid stools with or without blood within 24 hours, accompanied by dehydration, nausea, vomiting, abdominal cramping, fever, chills, muscle aches, and fecal urgency. A questionnaire that collected demographic information, illness onset and symptoms, medical care sought, and food/drink consumed was completed by the patient or a family member. Patients with other associated illness and who used antibiotic drugs before hospitalization were not included in this study. 

Fecal specimens were collected in McCartney bottles (KM Enterprises, Kolkata, India) by using sterile catheters or rectal swabs in Cary Blair medium (Difco, Sparks, MD, USA) and were examined within 2 hours for enteric pathogens comprising bacterial, viral, and parasitic pathogens by using a combination of conventional, immunological, and molecular methods (*16*). Patients were observed until their discharge from the hospital. The patients lived in different areas of the Kolkata Municipal region.

We screened for carbapenem resistance in multidrug-resistant isolates of diarrheagenic *E. coli*, *V. cholerae*, *V. parahaemolyticus*, *V. fluvialis*, *Salmonella* spp., and *Shigella* spp. isolated from these patients. We detected *V. fluvialis* and *bla*_NDM-1_ by using simplex PCR with previously described methods, lysed cells as templates (*17*,*18*), and Taq DNA polymerase (Roche, Mannheim, Germany). Amplicons were purified by using a QIAquick PCR Purification Kit (QIAGEN, Hilden, Germany) and sequenced by using the ABI BigDye Terminator v3.1 Cycle Sequencing Ready Reaction Kit (Applied Biosystems, Foster City, CA, USA) in an automated DNA sequencer (ABI 3730; Applied Biosystems). Sequences were edited with Lasergene software (DNASTAR, Inc., Madison, WI, USA) and analyzed by using BLAST (http://www.ncbi.nlm.nh.gov/blast).

We tested antibiotic susceptibility according to Clinical Laboratory Standards Institute (CLSI) guidelines (*19*) using commercially available antibiotic discs (Becton-Dickinson, Sparks, MD, USA) for ampicillin, cefuroxime, ceftriaxone, cefotaxime, cefotaxime/clavulanic acid, ceftazidime, ceftazidime/clavulanic acid, chloramphenicol, erythromycin, gentamicin, nalidixic acid, ciprofloxacin, ofloxacin, norfloxacin, imipenem, streptomycin, azithromycin, tetracycline, and trimethoprim/sulfamethoxazole. We used ceftazidime and cefotaxime to confirm production of extended-spectrum β-lactamase by double-disk synergy test. We determined MICs of imipenem, ciprofloxacin, norfloxacin, ceftazidime, cefotaxime, and cefepime using Etest strips (bioMérieux, Marcy l’Étoile, France) following the CLSI interpretive criteria for *Vibrio* spp. (*20*). For noncholera *Vibrio* spp., the CLSI guidelines lack interpretive criteria for some antibiotic drugs; hence, we used breakpoints for *E. coli* ATCC 25922, which was used as a control in antimicrobial drug susceptibility testing.

We performed the modified Hodge test on Mueller–Hinton agar (Difco) plates, using *E. coli* ATCC 25922 as the indicator organism and a 10-μg imipenem disk (*21*). The modified Hodge test is a phenotypic assay for the detection of carbapenemase enzyme–producing bacteria. This assay is based on the inactivation of a carbapenem by carbapenemase-producing test isolates that facilitate a carbapenem-susceptible indicator strain (*E. coli* ATCC 25922) to spread its growth toward a carbapenem-containing disc along the streak of inoculum of the test isolate. A positive test result produces a cloverleaf-like hollow.

We used the Kado and Liu method (*22*) to extract plasmid DNA from donors, recipients, and transconjugants and analyzed it by gel electrophoresis using 0.8% agarose. We used a PCR-generated DNA probe by the chemoluminescent method (ECL nucleic acid detection system; GE Healthcare Life Sciences, Buckinghamshire, UK) to make Southern hybridization to confirm the presence of *bla*_NDM-1_ in the plasmids. Plasmid-mediated transfer of antibiotic resistance from a NDM-1–positive *V. fluvialis* isolate (IDH 04744) to *E. coli* J53 (having dual resistant markers for nalidixic acid and sodium azide [Na-Az^R^]) was tested on MacConkey agar plates (Difco) containing sodium azide (100 mg/L) and meropenem (5 mg/L). Another plasmid-mediated transfer of antibiotic resistance from a NDM-1–positive *V. fluvialis* isolate (IDH 05720) has also been tested with diarrheagenic *E. coli*, *Salmonella* spp., and *Shigella* spp. and *V. parahaemolyticus* on meropenem (5 mg/L) supplemented MacConkey, xylose lysine deoxycholate, and thiosulfate citrate bile sucrose agar (Difco) plates.

Presence of *bla*_NDM-1_ in the transconjugants was confirmed by PCR. We used PCR and amplicon sequencing to identify other antibiotic resistance genes (*aadB*, *aadA1*, *strA*, *aphA1–1a*, *catA1*, *bla*_TEM-9_, *bla*_OXA-1_, *bla*_OXA-7_, *bla*_OXA-9_, *bla*_SHV_, *bla*_PSE-4_, *bla*_CTX-M-3_, *aac[6′]-1b-cr*, and *floR*) using lysed cells, primers, and previously described conditions (*23*). We used published primers to determine integrons and resistance gene cassettes in *V. fluvialis* isolates by PCR (*24*). The PCR amplicons were purified and directly sequenced. The identities of the sequences were established through a database search by using BLAST and matched with the reference *dfrA1* sequence of *V. fluvialis* (GenBank accession no. AY605688).

We determined the replicon types of *bla*_NDM-1_ harboring plasmids from the wild isolates and transconjugants by PCR using published methods (*25*). Sequencing of the *bla*_NDM-1_ and its flanking regions were made from a wild isolate of *V. fluvialis* (IDH 05720) by primer walking. The DNA sequence reported in this study has been deposited in GenBank (accession no. KR733543).

Pulsed-field gel electrophoresis (PFGE) analysis of *Not*I-digested genomic DNA of *bla*_NDM-1_–harboring *V. fluvialis* isolates (NDM-VF) was performed by using a CHEF-Mapper (Bio-Rad Laboratories, Hercules, CA, USA) according to the PulseNet standardized protocol for subtyping of *V. cholerae* (*26*). The PFGE image was captured by using a Gel Doc XR system (Bio-Rad). The PFGE image was normalized by aligning the peaks of the *Xba*I size standards of *Salmonella enterica* serovar Braenderup (H9182) in each gel and was analyzed by using BioNumerics software version 4.0 (Applied Maths, Sint-Martens-Latem, Belgium). The similarities between isolates were evaluated by using the cluster analysis with the UPGMA method and the Dice correlation coefficient with a position tolerance of 1.5%.

## Results

A total of 115 *V. fluvialis* were isolated from the acute diarrheal patients (each isolate represent a case), of which 27 (23.5%) were resistant for carbapenem and harbored *bla*_NDM-1_. The first *bla*_NDM-1_–positive *V. fluvialis* was isolated on May 16, 2011. The isolation rate of NDM-VF was highest in 2012 (14 isolates), followed by 7 in 2011 and 6 in 2013. The NDM-VF was not detected during 2009–2010. The rest of the pathogens tested in this study were susceptible to carbapenem.

Of the 27 NDM-VF strains, 13 (48.1%) were isolated as the sole pathogen; the remaining were co-pathogens isolated with any other pathogen, such as diarrheagenic *E. coli*, *Shigella* spp., *Salmonella* spp., *Campylobacter* spp., *Giardia lamblia*, and rotavirus. None of the enteric bacteria identified as co-pathogens had *bla*_NDM-1_. Most of the NDM-VF were resistant to ampicillin, ceftriaxone, cefuroxime, cefotaxime, nalidixic acid, norfloxacin, ciprofloxacin, ofloxacin, and streptomycin (100% each), followed by trimethoprim/sulfamethoxazole (96.2%), imipenem (88.8%), gentamycin (74.0%), chloramphenicol (70.3%), and tetracycline (14.8%). However, most of the isolates were susceptible to azithromycin (85%) and doxycycline (100%). Higher MICs were observed for cefotaxime (>16 mg/L), ceftazidime (>32 mg/L), cefepime (>16 mg/L), and cefotetan (>32 mg/L). In 50% of the NDM-VF, ciprofloxacin MIC was >32 mg/L, and for norfloxacin and imipenem the MIC values ranged from 4–32 mg/L (Table 1).

**Table 1:** Antimicrobial drug resistance genes and MICs of *Vibrio fluvialis* isolates in study of diarrheal fecal samples from patients in Kolkata, India, May 2009–September 2013*†

Isolate no.	Resistance gene profile	MIC, μg/mL							
		IPM	CTX	TAZ	PM	CIP	NOR	CN	TET†
IDH 03626	*bla*_NDM-1_*, bla*_TEM-9_*, bla*_CTX-M-3_*, bla*_OXA-1_*, bla*_OXA-7_*, bla*_OXA-9_, *aadA1, aadB,aac(6´)-Ib-cr, sul1*	8	>16	>32	>16	32	16	>32	ND
IDH 03631	*bla*_NDM-1_*, bla*_TEM-9_*, bla*_CTX-M-3_*, bla*_OXA-1_*, bla*_OXA-7_*, bla*_OXA-9_, *aadA1, strA, aadB, aac(6´)-Ib-cr, sul1, sul3, floR*	16	>16	>32	>16	32	16	>32	ND
IDH 03645	*bla*_NDM-1_*, bla*_TEM-9_*, bla*_CTX-M-3_*, bla*_OXA-1_*, bla*_OXA-7_*, bla*_OXA-9_, *aadA1, strA, aadB, aac(6´)-Ib-cr, sul1, sul3, floR*	16	>16	>32	>16	>32	48	>32	ND
IDH 03671	*bla*_NDM-1_*, bla*_TEM-9_*, bla*_CTX-M-3_*, bla*_OXA-1_*, bla*_OXA-7_*, bla*_OXA-9_, *aadA1, strA, aadB, aac(6´)-Ib-cr, sul1, sul3, floR*	16	>16	>32	>16	>32	16	>32	ND
IDH 03679	*bla*_NDM-1_*, bla*_TEM-9_*, bla*_CTX-M-3_, *bla*_OXA-1_*, bla*_OXA-7_*, bla*_OXA-9_, *aadA1, strA, aadB, aac(6´)-Ib-cr, sul1, sul3, floR*	16	>16	>32	>16	32	24	>32	ND
IDH 03893	*bla*_NDM-1_*, bla*_TEM-9_*, bla*_CTX-M-3_*, bla*_OXA-1_*, bla*_OXA-7_*, bla*_OXA-9_, *aadA1, strA, aadB, aac(6´)-Ib-cr, tetB, sul1, sul3*	4	>16	>32	>16	8	12	>32	24
BCH01733	*bla*_NDM-1_*, bla*_TEM-9_*, bla*_CTX-M-3_*, bla*_OXA-1_*, bla*_OXA-7_*, bla*_OXA-9_, *aadA1, strA, aadB, aac(6´)-Ib-cr, tetB, sul1, sul3*	32	>16	>32	>16	8	12	>32	24
IDH 04022	*bla*_NDM-1_*, bla*_TEM-9_*, bla*_CTX-M-3_*, bla*_OXA-1_*, bla*_OXA-7_, *bla*_OXA-9_, *aadA1, strA, aadB, aac(6´)-Ib-cr, sul1, sul3, floR*	8	>16	>32	>16	>32	24	>32	ND
IDH 04149	*bla*_NDM-1_*, bla*_OXA-1_*, bla*_OXA-7_*, bla*_OXA-9_*, aadA1, strA, aadB*, *aac(6´)-Ib-cr, sul1, sul3*	4	>16	>32	>16	8	12	>32	ND
IDH 04166	*bla*_NDM-1_*, bla*_TEM-9_*, bla*_CTX-M-3_, *bla*_OXA-1_*, bla*_OXA-7_*, bla*_OXA-9_, *aadA1, aadB, aac(6´)-Ib-cr, sul1*	8	>16	>32	>16	>32	16	>32	ND
IDH 04169	*bla*_NDM-1_*, bla*_TEM-9_*, bla*_CTX-M-3_*, bla*_OXA-1_*, bla*_OXA-7_*, bla*_OXA-9_, *aadA1, strA, aadB, aac(6´)-Ib-cr, sul1, sul3, floR*	16	>16	>32	>16	32	16	>32	ND
IDH 04228	*bla*_NDM-1_*, bla*_OXA-1_*, bla*_OXA-7_*, bla*_OXA-9_*, aadA1, strA, aadB*, *aac(6´)-Ib-cr, sul1, sul3*	8	>16	>32	>16	>32	32	>32	ND
IDH 04252	*bla*_NDM-1_ *, bla*_OXA-1_ *, bla*_OXA-7_ *, bla*_OXA-9_ *, aadA1, strA, aadB, aac(6´)-Ib-cr*	32	>16	>32	>16	32	24	>32	ND
IDH 04325	*bla*_NDM-1_ *, bla*_OXA-1_ *, bla*_OXA-7_ *, bla*_OXA-9_ *, aadA1, strA, aadB, aac(6´)-Ib-cr, sul1, sul3, floR*	8	>16	>32	>16	16	16	>32	ND
IDH 04326	*bla*_NDM-1_ *, bla*_OXA-1_ *, bla*_OXA-7_ *, bla*_OXA-9_ *, aadA1, strA, aadB, aac(6´)-Ib-cr, sul1, sul3, floR*	16	>16	>32	>16	>32	16	>32	ND
IDH 04382	*bla*_NDM-1_ *, bla*_OXA-1_ *, bla*_OXA-7_ *, bla*_OXA-9_ *, aadA1, strA, aadB, aac(6´)-Ib-cr, sul1, sul3, floR*	8	>16	>32	>16	32	16	>32	ND
IDH 04414	*bla*_NDM-1_ *, bla*_OXA-1_ *, bla*_OXA-7_ *, bla*_OXA-9_ *, aadA1, strA, aadB, aac(6´)-Ib-cr, sul1, sul3, floR*	8	>16	>32	>16	>32	32	>32	ND
BCH02360	*bla*_NDM-1_ *, bla*_OXA-1_ *, bla*_OXA-7_ *, bla*_OXA-9_ *, aadA1, strA, aadB, aac(6´)-Ib-cr, sul1, sul3, floR*	8	>16	>32	>16	32	24	>32	ND
IDH 04568	*bla*_NDM-1_*, bla*_TEM-9_*, bla*_CTX-M-3_*, bla*_OXA-1_*, bla*_OXA-7_*, bla*_OXA-9_, *aadA1, strA, aadB, aac(6´)-Ib-cr, tetB, sul1, sul3, floR*	32	>16	>32	>16	12	4	>32	16
IDH 04607	*bla*_NDM-1_ *, bla*_OXA-1_ *, bla*_OXA-7_ *, bla*_OXA-9_ *, aadA1, strA, aadB, aac(6´)-Ib-cr, sul1, sul3, floR*	32	>16	>32	>16	32	24	>32	ND
IDH 04744	*bla*_NDM-1_*, bla*_TEM-9_*, bla*_CTX-M-3_*, bla*_OXA-1_*, bla*_OXA-7_*, bla*_OXA-9_, *aadA1, strA, aadB, aac(6´)-Ib-cr, tetB, sul1, sul3, floR*	32	>16	>32	>16	>32	32	>32	24
IDH 05335	*bla*_NDM-1_*, bla*_TEM-9_*, bla*_CTX-M-3_*, bla*_OXA-1_*, bla*_OXA-7_*, bla*_OXA-9_, *aadA1, strA, aadB, aac(6´)-Ib-cr, sul1, sul3, floR*	8	>16	>32	>16	>32	24	>32	ND
IDH 05715	*bla*_NDM-1_*, bla*_TEM-9_*, bla*_CTX-M-3_, *bla*_OXA-1_*, bla*_OXA-7_*, bla*_OXA-9_, *aadA1, strA, aadB, aac(6´)-Ib-cr, sul1, sul3, floR*	2	>16	>32	>16	>32	32	>32	ND
IDH 05720	*bla*_NDM-1_*, bla*_TEM-9_*, bla*_OXA-1_*, bla*_OXA-7_*, bla*_OXA-9_, *aadA1, strA, aadB, aac(6´)-Ib-cr, sul1, sul3, floR*	8	>16	>32	>16	8	6	>32	ND
IDH 05733	*bla*_NDM-1_*, bla*_TEM-9_*, bla*_OXA-1_*, bla*_OXA-7_*, bla*_OXA-9_, *aadA1, strA, aadB, aac(6´)-Ib-cr, sul1, sul3, floR*	32	>16	>32	>16	32	24	>32	ND
IDH 05799	*bla*_NDM-1_*, bla*_TEM-9_*, bla*_CTX-M-3_, *bla*_OXA-1_*, bla*_OXA-7_*, bla*_OXA-9_, *aadA1, strA, aadB, aac(6´)-Ib-cr, sul1, sul3, floR*	24	>16	>32	>16	32	32	>32	ND
IDH 05818	*bla*_NDM-1_*, bla*_TEM-9_*, bla*_OXA-1_*, bla*_OXA-7_*, bla*_OXA-9_, *aadA1, strA, aadB, aac(6´)-Ib-cr, sul1, sul3, floR*	32	>16	>32	>16	32	32	>32	ND
*All isolates were positive in the modified Houge test and susceptible for imipenem with EDTA. CIP, ciprofloxacin; CN, cefotetan; CTX, cefotaxime; IPM, imipenem; ND, not done; NOR, norfloxacin; PM, cefepime; TET, tetracycline; TAZ, ceftazidime. †MIC assay was not done for TET-susceptible isolates.									

The *bla*_NDM-1_–harboring *V. fluvialis* isolates carried multiple plasmids ranging from 5 kb to 150 kb. In Southern hybridization, the large plasmids extracted from the transconjugants were positive for *bla*_NDM-1_. In the transconjugants, only a single plasmid of ≈80–90 kb was detected. The transconjugant (TC-J53) also showed resistance to ampicillin, erythromycin, streptomycin, ceftriaxone, cefotaxime, cefuroxime, and imipenem (MIC 2 mg/L), indicating the possibility that the NDM-1 plasmid also harbored genes encoding resistance to these antibiotics. The transconjugant was susceptible to ciprofloxacin, tetracycline, trimethoprim, chloramphenicol, and azithromycin, suggesting that the genes encoding resistance to these drugs are not carried by the *bla*_NDM-1_–harboring plasmid. Most of the other enteric pathogens used as transconjugants showed resistance to ampicillin, ceftriaxone, cefotaxime, and sulfamethoxazole. The transfer frequencies ranged from 1.4 × 10^3^ to 8.7 × 10^5^ (Table 2).

**Table 2:** Antimicrobial drug resistance profiles before and after transfer of NDM-1-–ncoding plasmid from *Vibrio fluvialis* strains to other bacterial species in study of diarrheal fecal samples from patients in Kolkata, India, May 2009–September 2013*

Isolate no.	Test isolate	Resistance profile of wild type	Resistance profile of transconjugant	MIC IPM, μg/mL†	Frequency of transfer
J53-Na-Azide	*Escherichia coli*	–	**AMP**,** CRO**, **SXT**, **CXM**, **CTX**	3	8.7 × 10^5^
BCH 04216	EAEC	AMP, ERY, SXT, NA, CXM, CTX	AMP, E, SXT, NA, CXM, CTX, **CRO**	3	5.9 × 10^5^
IDH 04184	EPEC	AMP, ERY, OFX, NOR, SXT, NA, CIP	AMP, E, OFX, NOR, SXT, NA, CIP, **CRO**, **CXM**, **CTX**	1.5	2.7 × 10^5^
IDH 06412	ETEC	AMP, ERY, NA, SXT	AMP, E, NA, SXT, CRO, **CXM**, **CTX**	3	7.5 × 10^5^
BCH 0704	*Salmonella* Bareilly	–	**AMP**, **CRO**, **SXT**, **CXM**, **CTX**	3	1.7 × 10^3^
570764	*Salmonella* Newport	–	**AMP**, **CRO**, **SXT**, **CXM**, **CTX**	3	1.4 × 10^3^
IDH 06370	*Shigella dysenteriae* 12	AMP, STR, NA	AMP, STR, NA, **CRO**, **SXT**, **CXM**, **CTX**	1.5	2.4 × 10^3^
IDH 06498	*Shigella flexneri* 4	AMP, E, STR, SXT	AMP, E, STR, SXT, **CRO**, **CXM**, **CTX**	3	4.4 × 10^3^
IDH 06342	*Shigella flexneri* 1b	AMP, E, NA, STR, SXT	AMP, E, NA, STR, SXT, **CRO**, **CXM**, **CTX**	0.75	1.9 × 10^3^
IDH 03988	*V. parahaemolyticus*	AMP	AMP, CRO, **SXT**, **CXM**, **CTX**	0.50	5.7 × 10^5^

*Antibiogram in bold among transconjugants denote acquired resistance phenotypes from *V*. *fluvialis* IDH05720 after conjugation. AMP, ampicillin; CIP, ciprofloxacin; CRO, ceftriaxone; CTX, cefotaxime; CXM, cefuroxime; EAEC, enteroaggressive *E. coli*; EPEC, enteropathogenic *E. coli*; ERY, erythromycin; ETEC, enterotoxigenic* E*. *coli*; NA, nalidixic acid; NDM-1, New Delhi metallo-β-lactamase; NOR, norfolxacin; OFX, ofloxacin; STR, streptomycin, SXT, sulfamethoxxozole. †MIC for imepenium. MIC denotes values of imepenium across different pathogens that were used as transconjugants.

Class-1 integron was identified in all the NDM-1–positive isolates. In 9 isolates, a 1.6-kb PCR amplicon was obtained with the dihydrofolate reductase gene cassette (*dfrA1*), which encodes resistance for trimethoprim. Overall, 9 different resistance gene profiles were identified (Table 1). All of the 27 NDM-VF isolates were positive for β-lactamase–encoding genes *bla*_OXA-1_, *bla*_OXA-7_, and *bla*_OXA-9_; streptomycin-encoding gene *aadA1*; gentamycin-encoding gene *aadB*; and ciprofloxacin-modifying enzyme-encoding gene *aac(6′)Ib-cr* (amino glycoside actetyltransferase)*.* Most NDM-VF isolates had *sul1*, conferring resistance to sulfonamides (96.2%); *strA*, conferring resistance to streptomycin (92.6%); and *sul3*, conferring resistance to sulfonamides (88.8%). The *floR* gene that encodes resistance to chloramphenicol was found in 20 (74%) of NDM-VF isolates. The other β-lactamase encoding genes, *bla*_TEM-9_ and *bla*_CTX-M-3_, which confer resistance to ceftriaxone, were detected in 15 (55.5%) isolates. The tetracycline resistance marker gene *tet(B)* was detected in only 4 isolates (14.8%).

**Figure 1:**
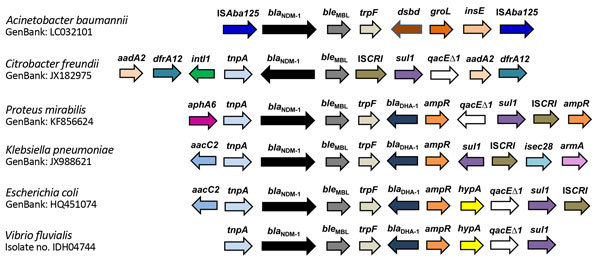
Structural features of *bla*_NDM-1_ flanking regions of *Vibrio fluvialis* and other bacterial species in study of diarrheal fecal samples from patients in Kolkata, India, May 2009–September 2013. Arrow lengths are proportionate to the lengths of the genes or open reading frames. GenBank accession numbers are shown. Gene names: *ISAba125*, insertion sequence *bla*_NDM-1_, New Delhi metallo-β-lactamase; *ble*_MBL_, bleomycin resistance protein; *trpF*, phosphoribosylanthranilate isomerase; *dsbd*, cytochrome c-type biogenesis protein; *groL*, chaperonins; *insE*, transposase insertion sequence; *aadA2*, aminoglycoside adenyltransferase; *dfrA12*, dihydrofolate reductase; *IntI1*, class I integron integrase; *tnpA*, transposition transposase; ISCR*I*, insertion sequence common region; *sulI*, dihydropteroate synthase; *qacE∆1*, ethidium bromide resistance protein; *aphA6*, aminoglycoside phosphotransferase; *bla*_DHA-1_, Class C β-lactamase; *ampR*, transcriptional regulator; *aacC2*, aminoglycoside acetyltransferase; *isec28*, transposase; *armA*, 16S rRNA methylase; *hypA*, putative hydrogenase nickel incorporation protein.

In replicon typing, plasmids of NDM-VF isolates were untypeable. To gain insight into the genetic background of *bla*_NDM-1_, the flanking regions of this gene were examined in a representative *V. fluvialis* isolate (IDH 04744). *bla*_NDM-1_ flanking sequences of IDH 04744 *V. fluvialis* were identical to the ones reported in the *E. coli* isolates from Hong Kong, China (pNDM-HK; GenBank accession no. HQ451074), and from a Spanish traveler returning from India (DVR22; GenBank accession no. JF922606.1) (Figure 1). The left junction of the sequences starts upstream of the *bla*_NDM-1_ with a truncated IS*Aba125* region, whereas the right junction possessed different genes such as *ble*_MBL_ (bleomycin-resistance encoding gene), *trpF*, *bla*_DHA-1_, and *ampR*.

**Figure 2:**
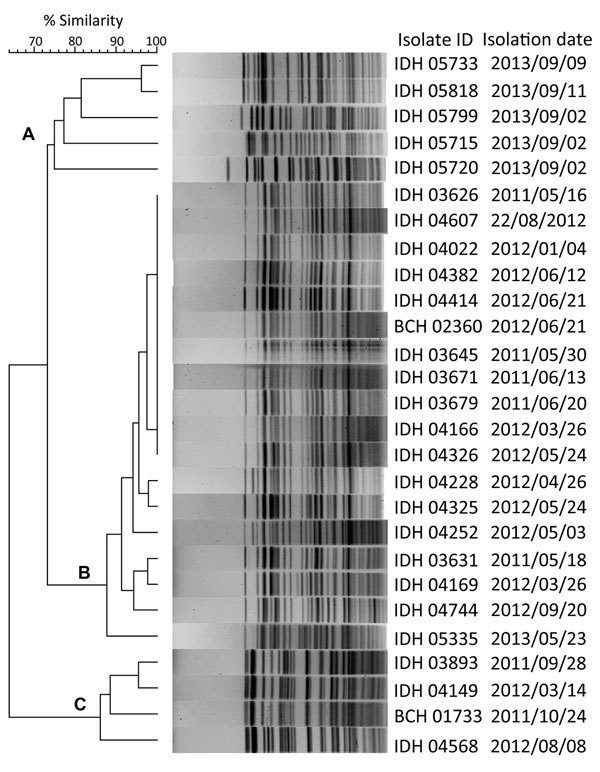
Pulsed-field gel electrophoresis analysis of *Not*I-digested genomic DNA of *bla*_NDM-1_ harboring *V. fluvialis* isolates in study of diarrheal fecal samples from patients in Kolkata, India, May 2009–September 2013. In the dendrogram, 3 distinct clusters (A–C) formed on the basis of the band similarity. Isolate identification (ID) includes name of associated hospital: IDH, Infectious Diseases Hospital; BCH, B.C. Roy Memorial Hospital for Children. *bla*_NDM-1_, New Delhi metallo-β-lactamase.

Eighteen different patterns that could be grouped into 2 distinct clusters (A–C; Figure 2) were obtained in the PFGE analysis. Most of the isolates in cluster B had ≈90%–100% similarity. Nearly identical PFGE profiles were obtained for 11 isolates (cluster B). These isolates were isolated over a span of 1 year (May 2011—May 2012), without any epidemiologic link. We also found no correlation between the PFGE and antimicrobial drug resistance patterns.

## Discussion

Since its discovery, global distribution of *bla*_NDM-1_ in different bacterial species has been extensively documented (*27*). NDM-1 producers are reported not only from patients epidemiologically linked to the Indian subcontinent but also from several indigenous cases all over the world with no such link. Previously, we reported on the emerging trend of *V. fluvialis* among the diarrheal cases in the Kolkata region (*14*). However, NDM-VF emerged in Kolkata during 2011, and 25%–50% of the *V. fluvialis* isolates harbored *bla*_NDM-1_ each year until 2013. It is difficult to epidemiologically link the isolates because of the wide difference in the dates of isolation of NDM-VF, lack of common food sources, and variation in the proximity of the residential area of the patients; antibiogram and PFGE patterns are also widely divergent.

NDM-1 producers have been found to be highly resistant to several classes of antibiotics (*28*–*30*), related to their unusual genetic assemblage, which helps in the acquisition and transfer of many resistance genes. Environmental strains of *Aeromonas caviae* and *V. cholerae* were found to carry *bla*_NDM-1_ on the chromosomes (*12*). In contrast, we found that, in NDM-VF, *bla*_NDM-1_ is present on the large plasmids.

Generally, the emergence of NDM-1 producers is associated with excessive use of carbapenems in patients with nonintestinal infections that necessitate a prolonged stay in a hospital. However, none of the patients in this study had a history of using carbapenem drugs. Most NDM-VF isolates remained susceptible to azithromycin, which is currently used in the treatment of diarrheal patients in Kolkata.

We found that a large plasmid from NDM-VF was effectively transferred to *E. coli* J53 and other enteric pathogens. Even though we demonstrated the in vitro transfer of *bla*_NDM-1_ in other enteric bacteria, these bacteria are not completely resistant to carbapenems, as is *V. fluvialis*. Multiple NDM-1–producing pathogens belonging to different species from a patient have been reported (*31*). Although in our study, 14 of 27 patients were infected with other pathogens (enteroaggressive *E. coli* [EAEC], enterotoxigenic *E*. *coli* [ETEC], *V. cholerae*, *V. parahaemolyticus*, *Salmonella* spp., *Shigella* spp., and *Campylobacter* spp.), only patients with *V. fluvialis* were found to harbor *bla*_NDM-1_. The controlling factors that may prevent such transfer in the gut milieu should be explored further.

The resistance profiles of ampicillin, ceftriaxone, trimethoprim/sulfamethoxazole, cefuroxime, and cefotaxime have been transferred to all the transconjugants. This indicates that the *bla*_NDM-1_–positive isolates may carry similar plasmids with the uniform resistance genes and, hence, confer the same resistance phenotype. Generally, the conjugative plasmids carrying *bla*_NDM-1_ have been classified into several replicon types, including IncA/C, IncFII_Y_, IncHI1b, IncX3, and IncT (*32*). However, the NDR-VF isolates were negative for all the NDM-1 plasmids in the PCR-based replicon typing. These results suggest that the NDM-1–encoding genes move with several plasmid scaffolds or as the same Inc type, which might not be covered by the currently used replicon typing scheme of *Enterobacteriaceae*. In many bacterial species from India, the *bla*_NDM-1_–harboring plasmids were found to belong to A/C-type, an uncommon group for conferring multidrug-resistant phenotypes (*3*).

Analysis of the genes adjoining the *bla*_NDM-1_ in *V. fluvialis* isolate IDH 04744 revealed a high homology with *E. coli* NDM-HK and DUR-22 (GenBank accession nos. HQ451074 and JF922606) (*33*,*34*). Insertion sequences (IS) IS*26* and IS*Aba125* have been identified upstream of the *bla*_NDM-1_ gene, and these sequences have been reported in other organisms. In most of the NDM-1–positive bacteria, the IS elements are detected in the flanking regions of *bla*_NDM-1_. We detected the IS*26* and IS*Aba125* in the upstream of the *bla*_NDM-1_ gene. The presence of IS*26*, IS*CR1*, and transposases have been increasingly implicated in interspecies and intraspecies dissemination of antimicrobial drug resistance genes (*35*,*36*). These IS elements probably help in the mobility of *bla*_NDM-1_. 

We also identified the *ble*_MBL_ gene downstream of *bla*_NDM-1_. In most of the *Enterobacteriaceae*, *bla*_NDM-1_ has been detected between a truncated IS*Aba125* located upstream and *ble*_MBL_ at the downstream. This genetic arrangement suggests an en bloc acquisition of *bla*_NDM-1_ and *ble*_MBL_ through the IS*Aba125*-related mobilization system. The presence of *ble*_MBL_ appears to be an added advantage to the *bla*_NDM-1_–positive bacteria, because both genes are expressed under the control of single promoter; therefore, the presence of *ble*_MBL_ may help the *bla*_NDM-1_–bearing plasmids to spread in other bacterial species (*37*). 

*V. fluvialis* is increasingly being detected in our setting and among diarrheal patients (*14*). These *V. fluvialis* isolates are capable of readily acquiring antibiotic resistance genes through mobile genetic elements (*38*). Our findings indicate that *V. fluvialis* might acquire the *bla*_NDM-1_ gene without any antibiotic selective pressure. This pathogen also has the potential to transfer this gene to other enteric pathogens. PCR-based identification of the NDM-1 regions in suspected pathogens will be very useful. The *V. fluvialis* isolates harboring *bla*_NDM-1_ are mostly susceptible to doxycycline and azithromycin. Considering the pathogenicity of *V. fluvialis* to humans and its ubiquitous presence in the environment, the need for constant monitoring of this *Vibrio* species is ongoing*.*
